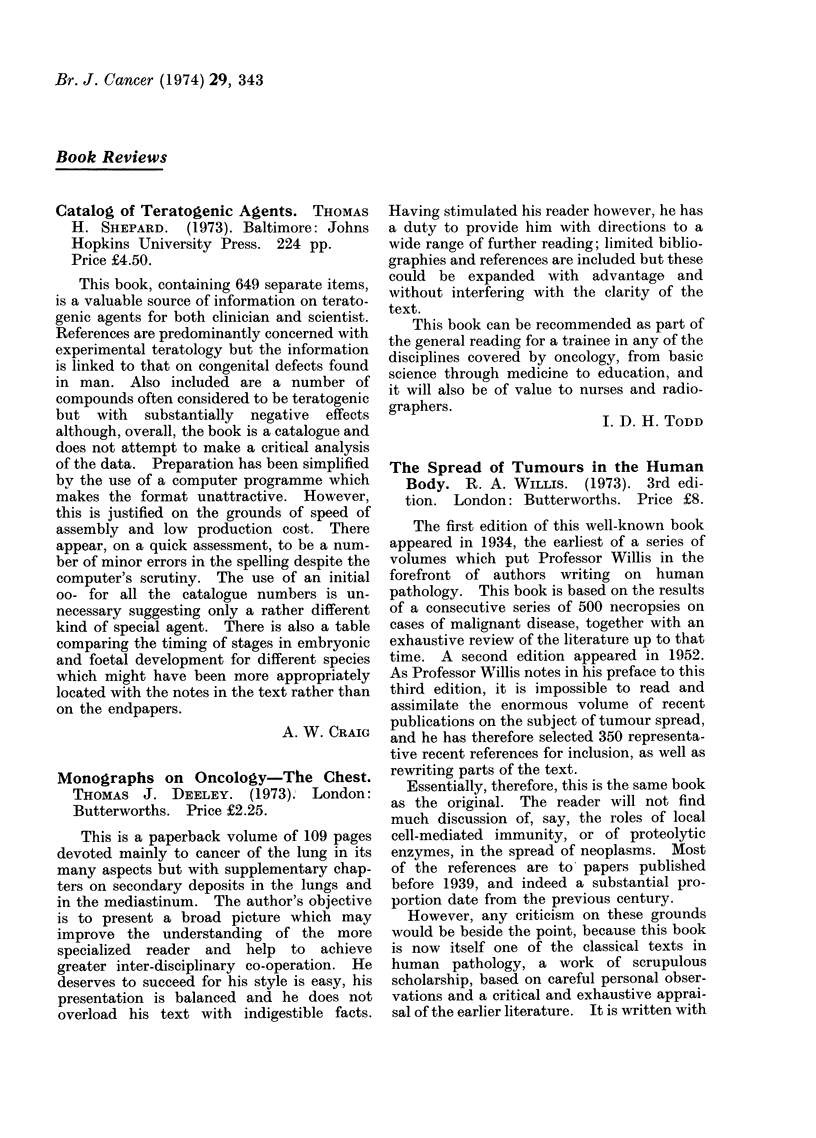# Catalog of Teratogenic Agents

**Published:** 1974-04

**Authors:** A. W. Craig


*Br. J. Cancer* (1974) **29**, 343

### *Book Reviews*

**Catalog of Teratogenic Agents.** THOMAS H. SHEPARD. (1973). Baltimore: Johns Hopkins University Press. 224 pp. Price £4.50.

This book, containing 649 separate items, is a valuable source of information on teratogenic agents for both clinician and scientist. References are predominantly concerned with experimental teratology but the information is linked to that on congenital defects found in man. Also included are a number of compounds often considered to be teratogenic but with substantially negative effects although, overall, the book is a catalogue and does not attempt to make a critical analysis of the data. Preparation has been simplified by the use of a computer programme which makes the format unattractive. However, this is justified on the grounds of speed of assembly and low production cost. There appear, on a quick assessment, to be a number of minor errors in the spelling despite the computer's scrutiny. The use of an initial oo- for all the catalogue numbers is unnecessary suggesting only a rather different kind of special agent. There is also a table comparing the timing of stages in embryonic and foetal development for different species which might have been more appropriately located with the notes in the text rather than on the endpapers.

A. W. CRAIG